# Review about Psychological Barriers to Lifestyle Modification, Changes in Diet Habits, and Health-Related Quality of Life in Bariatric Endoscopy

**DOI:** 10.3390/nu14030595

**Published:** 2022-01-29

**Authors:** Enrique Normand, Alejandro Montero, Gontrand López-Nava, Inmaculada Bautista-Castaño

**Affiliations:** 1 Bariatric Endoscopy Unit, HM Sanchinarro University Hospital, 28050 Madrid, Spain; monbelan@hotmail.com (A.M.); glopeznava@digestivolopeznava.com (G.L.-N.); inmaculada.bautista@ulpgc.es (I.B.-C.); 2 Centro de Investigación Biomédica en Red Fisiopatología de la Obesidad y la Nutrición (CIBEROBN), Instituto de Salud Carlos III, 28029 Madrid, Spain; 3 Research Institute of Biomedical and Health Sciences (IUIBS), University of Las Palmas de Gran Canaria, 35016 Las Palmas de Gran Canaria, Spain

**Keywords:** obesity, bariatric endoscopy, psychology, lifestyle and quality of life

## Abstract

Obesity is an expanding disease responsible for significant deterioration in the Health-Related Quality of Life (HRQL) of those who suffer from it. Bariatric Endoscopy (BE) therapies have proven to be an effective treatment for this pathology. A multidisciplinary approach is essential for the successful therapeutic management of BE. This article addresses the multidisciplinary treatment of BE by considering the possible variables that can influence treatment. In particular, the variables that can facilitate or hinder changes in patients’ habits are discussed. These include the neuropsychological, emotional, and social implications that may influence the formation of healthy habits necessary for improvement in a patient’s quality of life; the individual and environmental psychological factors that influence the monitoring of nutritional and physical activity indications; and different psychological disorders such as depression, anxiety, or disorders related to eating. The main objective of BE treatment, except in certain special biological situations, must be to establish a long-term sustainable change in habits such that patients, once they reach a healthy weight, do not revert to the lifestyle that caused their obesity, as well as identifying and addressing major problems that may exist prior to, or arise during, treatment.

## 1. Introduction

Obesity is a growing disease in Western countries. According to the WHO, 13% of the world’s population suffered from obesity in 2016 [1]. Furthermore, obesity prevalence is increasing rapidly in certain populations, with increases of up to 12% over a 3-year period (2013–2016) among certain groups [2].

Obesity is not only a disease in and of itself but is also a risk factor for multiple secondary diseases [1,3]. In addition to the health problems experienced by sufferers of obesity, numerous related social and cultural problems exist [4].

Traditional treatments based on diet and nutritional advice have proven ineffective in the treatment of obesity [5]; however, bariatric endoscopic (BE) treatments have yielded improved results [6,7,8,9]. Consequently, BE is postulated to be a less invasive alternative to surgical interventions and useful for high-risk morbidly obesity [10]. Scientific interest in these treatments has increased over recent years, with some studies employing these techniques demonstrating effective weight control up to 5 years later [6]. In relation with the rates of adverse events after BE techniques, serious side effects with the Orbera Intragastric Balloon were rare, with an incidence of migration and gastric perforation of 1.4% and 0.1%, respectively [11], and with Endoscopic Sleeve Gastroplasty and the POSE procedure [12,13], only a limited number of complications have been reported (postprocedure bleeding, perigastric collection with pleural effusion, perforation of the stomach, pneumothorax, and perihepatic or perisplenic abscess. No patient required an emergency intervention, and there were no mortalities. BE therapies such as endoscopic sleeve gastroplasty have been shown to be highly effective in weight loss in patients with obesity [7,8,9,10,13,14], increasing the feeling of satiety on the part of the patient physically. However, to achieve maximum effectiveness in this type of technique, multidisciplinary intervention by specialists in nutrition and psychology is required [15,16] as well as incorporating or increasing physical activity.

Obesity represents a risk factor for various cardiovascular diseases including hypertension, atherosclerosis, and myocardial infarction [17]; metabolic complications including type 2 diabetes, fatty liver disease, and different cancers; mechanical complications including joint pain and arthritis; increased obstetric risk; and sleep apnea. Additionally, obesity may adversely affect mental health, partly due to social stigmatization [18].

In addition to the several pathologies mentioned above, obesity is associated with deterioration in the perceived quality of life. Mobility problems, reduced self-care, a decreased ability to carry out daily activities, pain, and discomfort are some of the problems suffered by people with obesity [19]. In previous studies, there were over 181 patients who underwent EBT in a standardized multidisciplinary follow-up program, and we showed that at 9 months, the weight lost and the increase in physical activity improved HRQL measured by the Short Form-36 health survey with the physical (PSC) and mental (MSC) summary component scores to capture generic HRQL. The mean percentage of total body weight loss achieved after the intervention was 16.9 (9.7)% [20].

There are several automatic psychological mechanisms that influence the behavior and could be negative for treatment goals. Undergoing an intervention with the aim of losing weight alters automatic psychological mechanisms but with the passage of time will tend to recover the pre-existing mechanisms. Then, the unconscious tendency will be to recover the harmful habits that lead to being overweight. Therefore, it is essential to support the patient in this follow-up to achieve a change in their habits for a sufficient time and maintain the change permanently [21].

Most of the information included in this review manuscript is derived from the clinical experience included in the follow-up program of a BE unit with long experience in the multidisciplinary follow-up of patients with BE, mainly by the team’s psychologists.

## 2. Modification of Habits in Obesity

Obesity is a complex and multicausal disease associated with more than one hundred and thirty biological, environmental, behavioral, and psychological factors [4]; therefore, approaches to its treatment must be holistic and consider a variety of different perspectives. In the case of people with obesity, changing habits can be more complicated than in the population without obesity. The following section describes some of the psychological, neuropsychological, and environmental characteristics that can make it difficult to change habits in the population with obesity.

### 2.1. Executive Function Problems 

One approach to obesity treatment that is often overlooked is the neuropsychological approach. In regard to forming new habits and improving quality of life, patients with obesity have presented some characteristics that are of particular interest to clinicians.

The population with obesity shows impaired performance in some executive functions compared with healthy-weight individuals. Specifically, worse performance has been observed in planning capacity, inhibition capacity, cognitive flexibility, and delaying gratification, among others [22,23,24,25,26,27,28]. Deficits in these brain functions could be realized as difficulties in forming and maintaining new life habits, delaying gratification, and inhibiting oneself in the face of possible temptations associated with relapse into harmful routines. It has also been shown that the population with obesity perform worse in behavioral inhibition tasks; therefore, they may be less able to control their actions and may be more impulsive [24].

### 2.2. Food Addiction

Another factor that could make the alteration of eating habits more difficult is the possible existence of food addiction. The addictive consumption of substances is defined as a pattern of abusive and continued consumption of substances over time, regardless of the harmful consequences that such substances may have for the individual, in addition to an apparent loss of control over the addictive behavior. In other words, people continue to consume the substance even after experiencing the harmful consequences it can have on their health. In addition, this type of behavior is often accompanied by multiple unsuccessful attempts to stop or reduce consumption. From a neuropsychological perspective, this type of behavior points to possible alterations in inhibitory control and decision-making abilities [29].

Some researchers consider food addiction to be the same as any other addiction. This theoretical approach is controversial and is currently the subject of debate. Some of the arguments in favor of this approach are based on the fact that the same executive dysfunctions—including greater impulsivity in decision making, alterations in the frontal areas of the brain, alterations in areas of the limbic system, and attentional biases toward stimuli related to their pathology—have been observed in both population with obesity and populations with other addictions [30]. However, not all patients with obesity are food addicted; it is estimated that this “addiction” occurs in about 25% of the population with obesity [31]. If we translate these results into clinical practice, we would be faced with a scenario in which one out of every four patients with obesity would possess limited capacity to make adequate decisions due to excessive attention toward food that is harmful to their health and an altered brain reward system. As such, these results should be extrapolated with caution; however, they provide a perspective that clinicians dealing with this pathology could consider in the management of obesity.

### 2.3. Obesogenic Environment

According to the Cognitive Analytical Psychotherapy model, it is necessary to know the relational style of patients when making a psychological approach to the problem [32]. Therefore, when faced with the problem of obesity, it is necessary to consider the dynamics that the subject establishes with the environment in which they interact and how these dynamics influence them. 

It is well known that some environments may promote obesity more than others [4]. In Western society, there exists an environment that promotes the immediate consumption of easily accessible products [33]. This external influence can lead to or facilitate people’s consumption of unhealthy foods. In such scenarios, it is vital to form healthy habits, forge a sufficiently solid conviction to resist temptation, and to consider relapse prevention as part of the treatment itself [34,35].

Social context and the habits that can be acquired from the family environment are also extremely important factors in developing and remaining obesity [4]. Families with overweight or parents with obesity are more likely to have children with obesity. 

Genetics are one of the biggest factors examined as a cause of obesity. Some studies have found that BMI is 25–40% heritable. However, genetic susceptibility often needs to be coupled with contributing environmental and behavioral factors in order to affect weight. The genetic factor accounts for less than 5% of cases of childhood obesity [36].

It was observed that the children raised by mothers with obesity or overweight were between 4.5 and 6.5 times more likely to suffer from obesity than children with mothers of healthy weight [37]. Thus, seemingly insignificant family or leisure habits—such as rewarding good behavior with food, being forced to eat large meals, an obligation to finish all of the food that has been prepared, etc., can result in harmful internalized habits in adulthood. Breaking these types of associations alone is complex, since the strength of the associations has often grown since childhood, and many times, the adult is hardly aware of having internalized them or of how problematic they can be. Along these lines, changing the form of leisure time or the way in which people interact with their peers has been shown to yield positive weight loss results [38]. For this reason, one possible objective of an intervention team could be to identify and attempt to modify these harmful leisure time or relationship habits. 

## 3. Habit Change and BE

Obesity is known to be a multicausal disease [4] that requires multidisciplinary treatment [11]. BE treatments have demonstrated their efficacy in changing the HRQL of patients [14,22]. Therefore, it is important to highlight the need for a multidisciplinary approach to achieve long-term changes in habits and thereby avoid relapses. Motivation is a fundamental factor in achieving lasting change in the treatment of obesity. Maintaining motivation throughout the entire weight loss process is fundamental, since a decrease in motivation can facilitate the abandonment of acquired healthy habits and, consequently, the regain of weight.

In addition to the biological factors that can contribute to weight regain, behavioral and psychological factors must be considered in view of possible relapses in obesity [39].

In our clinical practice, postprocedure care with a nutritionist and a psychologist weekly or biweekly was maintained post BE procedures during 1 year. The psychologist coached patients to follow the recommended lifestyle modification program necessary to maintain their weight loss over the long term. Furthermore, patients were coached on how to interact with food cues and obesogenic environment stimuli. Finally, they were taught how to recognize emotional eating cues and deal with them [16]. 

During the treatment process, it is important that patients acquire a healthy focus on what the goal of treatment is. It is common for treatments limited to addressing the diet alone to result in regaining the lost weight after a significant weight loss process [40]. Patients sometimes achieve significant weight loss before relapsing into old habits and returning to an obese state [41].

When starting a BE process, it is essential to have adequate nutritional and psychological support [14]. Clinical experience tells us that in the early stages of such treatment, the patient has the possibility of reconfiguring their eating and behavioral habits. In the first month following the procedure, the patient is forced to eat in a healthier way if they do not want to experience punishment in the form of gastralgia, heavy digestion, feelings of guilt, etc. The interior-modifying effect of this potential initial discomfort can be very useful when forming new eating habits. At the beginning of the treatment, the patient must be aware of what they are eating, the quantity, the speed at which they ingest the food, the foods they can and cannot eat, etc. In this initial phase of treatment, the successes that are achieved, the hope for change, the fear of physical punishment (gastralgias) or emotional punishment (feelings of guilt, frustration, anger, or regret), and the fear of treatment failure all make it easier for the patient to form new lasting habits rather than simply changing their eating patterns on an occasional basis—as is usually the case in the habitual dieting process [5]. 

Another fundamental variable that should be addressed and maintained is the forming of physical activity habits in cases where they had not been formed beforehand. Changes in physical activity should be considered a necessary element of treatment when adapted to the individual’s capabilities. It is common for patients to adopt a passive attitude toward physical activity at first but, in the long term, increases in physical activity usually lead to positive treatment results [38].

It is not uncommon during treatment for patients to experience a sense of “revelation” about how harmful their previous behaviors were and how much they have changed. This stage is a suitable time for patients to internalize new eating habits such as being aware of when they are full and when they are satiated, understanding under what circumstances they are most prone to breaking their diet, and, if necessary, modifying their exposure to those circumstances.

### 3.1. The Usefulness of Guilt

Another fundamental habit to develop is acknowledgement of the usefulness of punishment. One of the emotions most commonly seen in people who require BE intervention is guilt. People may feel guilty for a variety of reasons: for having skipped or broken a diet, for needing help, for not meeting the hypothetical expectations of the therapeutic team, etc. At this point, it is essential that patients develop the ability to learn from guilt. Guilt teaches people that they have done something they regret; therefore, it can be useful in identifying certain harmful habits. On many occasions, patients are not able to identify the reason for their diet transgressions, but they are able to recognize when they have felt guilty about them. It has been shown that there are certain patterns, environments, or situations that facilitate dietary lapses [42]. Using guilt as a method of identifying and modifying these tendencies can facilitate interventions and increase patients’ sense of agency. However, clinicians must be aware of the intensity and consequences of these feelings of guilt, as an excess or mismanagement of this emotion can lead to binge eating or treatment abandonment.

### 3.2. The Usefulness of Fear

Another fundamental factor that contributes to changing habits in BE treatments is fear. Patients may experience fear of going back to the starting point or the fear of spoiling the procedure. It is important for people to learn how to handle these fears in a healthy way by using the energy that fear brings them to break the habits that led them to obesity in the first place and to form new habits that protect them from experiencing these fears. The long-term maintenance of acquired habits is considered a fundamental variable in the prevention of weight regain; therefore, maintaining motivation—either by acknowledging the success achieved or the fear of regression—can be of crucial importance to treatment success. As with guilt above, clinicians should be especially aware of the potential consequences of fear and try to reduce or control it in cases where restrictive processes, obsessions, or hypervigilance processes arise. 

Although, as previously mentioned, the stigma of obesity is not beneficial to its treatment and can be associated with emotional problems [43]; the possibility of leaving this stigma behind and the social reinforcement that people get from it are important components in maintaining healthy lifestyle habits. Psychological support at this point of treatment often consists of helping people to validate these emotions of fear, guilt, or rage, and to use them productively, rather than become trapped by them. The goal of such support is that the emotions that people have spent so much time feeling or hiding from themselves become useful in both the changing process and in maintaining a healthier and more satisfying life. 

### 3.3. Long-Term Reinforcement

Similarly, many patients receive positive reinforcement from their social environment as they lose weight or reach a healthy weight. Validating this reinforcement can be helpful in maintaining positive motivation during the early stages of treatment. Clinicians must be careful that patients do not become dependent on this reinforcement, as it is a reward that is destined to fade away. Individuals are highly gratified by external praise or encouragement as they achieve treatment goals. These external reinforcers are received less frequently as the person approaches the maintenance stage. Therefore, it is essential that during these stages, intrinsic motivation is developed, and the patient seeks to find and validate other sources of reinforcement. In this way, the patient will be able to face maintenance in an independent way and avoid relapses due to a lack of external reinforcement.

## 4. Adherence to Treatment

Different studies agree that psychosocial factors that negatively affect adherence to treatment in the overweight and obese are mainly the lack of social support and personality factors such as lack of self-control, lack of motivation, and negative mood [44].

Moreover, the lack of support from family and friends adversely affects patient adherence to treatment. Likewise, the lack of support from medical staff by patients being treated has a negative implication in the success of the treatment.

In this regard, several studies suggest the type of monitoring provided by medical staff is key because it is more beneficial for patients’ positive support; this is based on strengthening and active stimulation versus limited follow-up with verbal instructions [45,46,47]. In a study of overweight and women with obesity, it was found that adherence to the nutritional program was higher in women who received psychological follow-up compared to those who did not have follow-up or any other support [48].

In this sense, the role of the psychologist as part of the follow-up will be fundamental not only to support the patient but also to work on motivation and adherence to changes in their lifestyle through the application of psychoeducation and cognitive–behavioral techniques [49], anticipating barriers related to their personality and mental health that can negatively influence the achievement of objectives. The barriers to weight loss in the follow-up of patients operated on at the Bariatric Endoscopy Unit of the HM Sanchinarro University Hospital in Madrid, directed by Dr. López-Nava, are described below in more detail.

## 5. Difficult Situations to Lose Weight

Sometimes, the patient presents certain barriers related to their mental health prior to the intervention, although these can also occur during the follow-up. If we review the literature in this regard, we find that the most described mental disorders that affect or may affect weight loss on an ongoing basis are depressive disorders and anxiety disorders.

In a longitudinal study [50], it was observed that patients diagnosed with depression or anxiety who underwent bariatric surgery lost significantly less weight compared to those without this pathology. These results underscore the importance of addressing latent anxiety and depressive disorders in overweight patients or patients with obesity, especially when these patients undergo bariatric surgery.

A possible justification for this worse result is that there are different foods (such as carbohydrates) that are more stimulating to a patient with depression, since their intake triggers an increase in serotonin levels. For this reason, the brain preferentially seeks these foods that contain the components necessary to increase serotonin levels [51].

Similarly, in anxiety disorders, the patient has been able to condition eating behavior as a strategy to reduce anxiety levels, tending to choose higher calorie foods. Sometimes, the patient ingests large amounts of food with anxiety in a short time. This can trigger Feeding and Eating Disorders such as Binge-Eating Disorder or even Bulimia Nervosa, as described in the DSM-V [52]. Some studies show that Binge-Eating Disorder persists after being subjected to bariatric surgery techniques. According to a study carried out on 120 patients undergoing bariatric surgery, it was observed that four out of nine patients who binged before surgery continued to do so afterwards [53]. A follow-up study of 66 morbidly patients with obesity who underwent laparoscopic adjustable gastric band surgery showed that 24.2% of patients persisted with binging one year after the intervention [54]. The pervasiveness of binge-eating behavior may be due to hunger caused by food restriction, which together with anxiety causes a lack of control, disinhibition, and overeating.

Finally, evidence has been found that Attention-Deficit/Hyperactivity Disorder (ADHD) is related to obesity, since the mutation of the gene that encodes the MC4R protein (C271R) that causes obesity is also associated with ADHD [55]. The inhibitory control dysfunction present in patients with ADHD alters executive functioning, generating distraction, disorganization, and low tolerance for frustration, which is related to non-compliance with healthy lifestyle habits essential for weight loss. Furthermore, ADHD–obesity comorbidity increases the probability of binging compared to patients with obesity without ADHD [56].

## 6. Main Psychological Barriers That Influence Weight Regain

Hitherto, we have seen various altered psychological patterns that negatively impact the achievement of goals related to weight loss. However, there are also everyday, non-pathological causes drawn from professional practice that make weight loss difficult in patients undergoing BE treatment. It is not a question of unwillingness or reluctance to follow a diet and exercise, but rather, these difficulties are related to cognitive distortions or sabotaging thoughts that precede unwanted behaviors.

**Figure 1:**
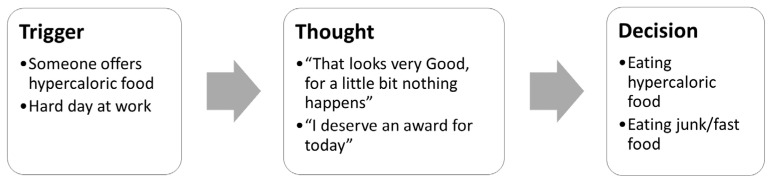
Examples of the consequences of sabotaging thoughts for a weight loss patient.

According to the cognitive–behavioral model, any situation causes the appearance of a thought after which the behavior appears, in this case, eating. This explains why people do not react in the same way to the same situation; it is the thoughts that determine the subsequent behavior and not the situation that triggers the act of eating. As an example, Figure 1 depicts two chains of events that patients frequently report.

These decisions produce immediate positive reinforcement in the patient, which is determined by their reward system, but in turn a negative emotion of guilt, disappointment, or frustration. Since we are talking about food, stopping triggers can be very difficult to prevent by having to eat several times a day and be exposed to food repeatedly throughout the day. For this reason, sabotaging thoughts are going to play an important role in the day-to-day life of patients.

### 6.1. Difficulty following Nutritional Guidelines

As already mentioned, one of the most recurrent psychological barriers in patients to follow nutritional guidelines is anxiety. Some patients have unconsciously learned over time to reduce their anxiety levels by eating. They tend to report more hunger in the afternoon, once they get home after a long working day during which they have had a busy mind. However, when they get home, sabotaging thoughts begin to appear, which are followed by grazing behaviors to neutralize that anxiety. The truth is that this does not work, because although the anxiety disappears momentarily, an emotion of guilt and disappointment emerges for their actions. Sometimes, even the patient connects this situation with new saboteur thoughts that encourage this unhealthy behavior, justifying initiating it and taking advantage of the occasion. This leads the patient to enter a downward spiral of negativity and an exponential rise in feelings such as guilt or hopelessness.

On the other hand, there are cases in which the patient tends to have an external locus of control, attributing the causes of his weight gain to factors unrelated to his behavior. This is supported by studies that show that an internal locus of control predicts a lower percentage of bariatric patients reneging, acting as a protective factor against patients with an external locus of control [57]. In practice, these patients usually have the belief that they are following the guidelines correctly but that they are not losing weight because the bariatric intervention has not been performed or has not been successful, or they have cognitive distortions related to a negative vision of the world such as bad luck or everything is against you when in fact they are not aware that they are not following the nutritional guidelines correctly. Sometimes, the key lies in the return to inappropriate behaviors such as snacking, nocturnal, and emotional eating, disorder in mealtimes, incorporating non-regulated foods in the diet, and improvising the menu.

Purely behavioral factors such as speed of ingestion represent a barrier for a large percentage of patients. The problem is that patients who eat too fast, in less than 20 min, do not give enough margin for their hypothalamus to stop the neurological signal of hunger and therefore tend to eat more. In some patients, in addition, personality traits of neuroticism and impulsivity predominate, which negatively affect the mediating responses of food restriction and emotional intake to external signals.

All of this can be reinforced by environmental factors, as is the case of patients who due to the characteristics of their work are forced to eat in restaurants or eat in a very short time frame. However, to a greater or lesser extent, it will always be ensured that these patients adapt and anticipate these types of situations, even restructuring the nutritional plan so that this does not affect their weight loss. Other environmental factors are those related to the occupation of the patient. In gastronomic trades, this poses a difficulty in adapting to new habits, since they are very frequently exposed to food and are even in charge of cooking and tasting dishes due to the nature of the job. However, there are patients who despite these difficulties are able to change habits and lose weight—so in no case is it a determining factor of non-compliance with nutritional guidelines.

**Figure 2:**
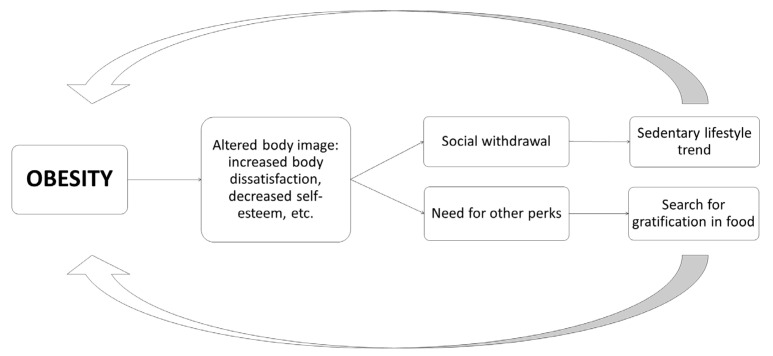
Example of how a consequence of obesity in the psychological field can enhance it [58].

Finally, it is worth highlighting the influence of the patient’s self-esteem and self-image as they progress in weight loss. Although the loss of volume is usually one of the main factors that reinforce the patient and motivate him to continue the process, sometimes, difficulties arise, since the patient does not always adapt to his new body image, especially due to the excess skin and the influence of unrealistic expectations, which may even cause sexual difficulties, among other narratives. As reflected in Figure 2, the alternation of body image can become a barrier that enhances weight regain [58].

### 6.2. Difficulties Doing Physical Activity

The incorporation and maintenance of physical activity in the weight loss process is crucial not only because it affects the energy balance but also because it moderates appetite and helps control emotional states of stress and anxiety. Thus, physical activity can prevent the intake of hypercaloric foods by calming the nerves in the short term [59].

As with nutritional guidelines, many patients look for reasons to justify the difficulty in not exercising. In this regard, patients also tend to use an external locus of control, narrating that it is impossible for them to acquire an exercise routine due to the daily hours they dedicate to work, having to take care of their children or dependent parents, not having a nearby space in which to exercise or even because the weather conditions are adverse. However, these reasons are not direct causes of sedentary lifestyle but rather alibis, since, although they are factors that make it difficult to incorporate exercise as a daily routine, they do not make it impossible. For example, although the rain may seem like a reason not to go outside to exercise, the reality is that it is not the cause of not doing physical activity, since you can adapt by doing it at home. Patients sometimes fall into certain sabotaging thoughts that break their commitment, which weakens their personal self-control and makes them more likely to fall into a sedentary lifestyle.

**Figure 3:**
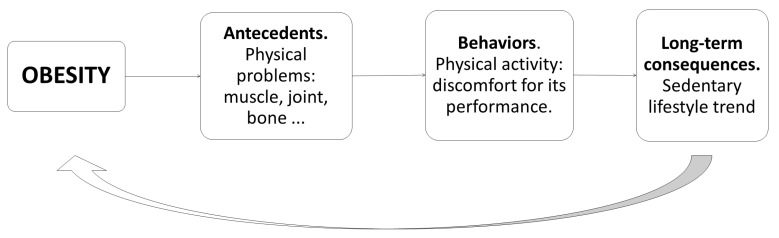
Example of how a consequence of obesity in the physical field can enhance it [58].

Sometimes, the basic problem is that the patient generates unrealistic expectations, setting goals that are too ambitious, which is counterproductive, since exercise in patients with obesity initially causes exhaustion due to overexertion. All this contributes to the fact that after exercising, the patient has negative sensations or even injuries and pain, which can generate aversion to physical activity and therefore discourage performance in the future. This contributes negatively to weight loss and therefore enhances the circle, as can be seen in Figure 3 [57].

## 7. Abandonment of the Follow-Up Schedule and Consequences

As already mentioned, different studies highlight the importance of making follow-up appointments for patients with obesity to increase their chances of success, taking into account previous difficulties or difficulties that may arise throughout the therapeutic process [15,16,45,46,47,48,49].

In this sense, a complete study carried out on 962 patients with obesity treated with endoscopic bariatric therapies concluded that weight loss one year from the date of intervention did not depend so much on the type of procedure as on the follow-up assistance by the patient [16]. The multidisciplinary follow-up program for this study consisted of nutritional instructions, psychological support, physical activity, and a planned counselling program, as well as a schedule for future visits. Frequent interaction with the patient may have provided the opportunity to identify “at risk of failure” patients and intervene early. In addition, the psychological counseling and support received were able to promote sustained weight loss. Unlike bariatric surgical patients, BE therapies patients are more motivated to achieve results with a less invasive treatment option. However, half of the patients in this study were lost to the one-year follow-up. Most of these patients were unmotivated, as they did not achieve a significant weight loss per month.

Despite its importance, poor adherence and loss to follow-up remain an unsolved problem with many obesity treatments [60,61].

## 8. Conclusions

Obesity is a disease that deteriorates the HRQL of those who suffer from it. Some characteristics of this pathology prevent patients from making the changes to vital habits that are necessary to promote and maintain a good quality of life.

BE treatments are an effective treatment for obesity. This therapeutic approach has the advantage of being less invasive than other surgical approaches and more effective than other more traditional treatments. 

The single combination of diet and exercise is not enough to achieve effective and sustained weight loss for the patient as well as prevent long-term weight regain. It is necessary to combine it with integrated psychological intervention in the multidisciplinary work of a healthcare team that contributes to an active and stimulating monitoring of the patient.

Given the multicausal components of obesity, BE treatment must take a multidisciplinary approach to this complex problem. Among the objectives to be achieved, one of the most important must be the establishment of new lifestyle habits that contribute to weight loss and the maintenance of a healthy weight.

Evidently, the importance of carrying out a psychological evaluation of patients who undergo bariatric endoscopy techniques helps identify psychological disorders such as depression or anxiety, which may affect the subsequent evolution of the patient. In this way, these problems can be accounted for through a subsequent psychological intervention based on cognitive restructuring, relapse prevention, and reinforcement in the achievement of the patient’s goals in their weight loss process.

BE units must note that this approach to obesity treatment should consider a variety of specific factors. Increasing inhibition capacity, improving decision-making abilities, and establishing an internal locus of control are some of the many variables to be considered. 

**Figure 4:**
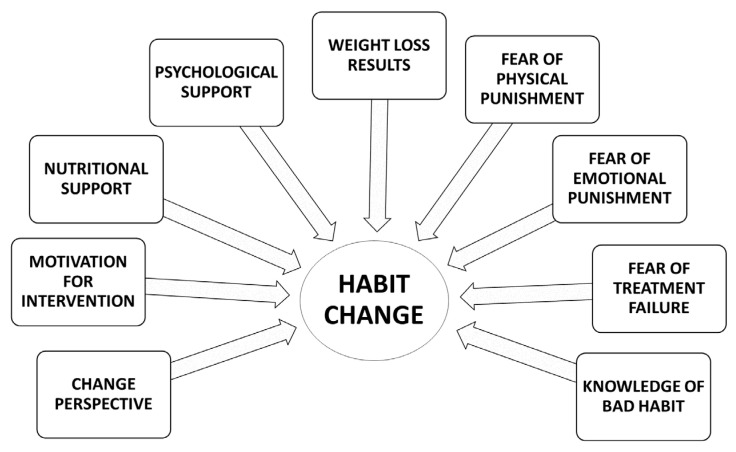
Support factors and motivation for change in bariatric endoscopy.

Forming healthy and protective habits against possible relapses must constitute another central objective of the intervention. Planning for and acknowledging the usefulness of relapses as part of the treatment course can be of great help to patients when attempting to consolidate long-term improvement. Likewise, using the components of BE treatments to strengthen the establishment of new habits and improve quality of life can be useful for professionals. In this study, the following characteristics of BE were identified as strengths or “motivational opportunities”: change of life perspective, motivation for intervention, nutritional support, psychological support, weight loss results, fear of physical punishment, fear of emotional punishment, fear of treatment failure, and knowledge of bad habits (Figure 4). These strengths should be used to form new life habits and in this way improve the long-term HRQLe of patients with obesity.
